# Impact of community engagement and social support on the outcomes of HIV-related meningitis clinical trials in a resource-limited setting

**DOI:** 10.1186/s40900-020-00228-z

**Published:** 2020-08-20

**Authors:** Richard Kwizera, Alisat Sadiq, Jane Frances Ndyetukira, Elizabeth Nalintya, Darlisha Williams, Joshua Rhein, David R. Boulware, David B. Meya, on behalf of the COAT and ASTRO trial teams, David B. Meya, Abdu Musubire, Henry W. Nabeta, Andrew Kambugu, Yukari C. Manabe, Jane Francis Ndyetukira, Cynthia Ahimbisibwe, Florence Kugonza, Alisat Sadiq, Richard Kwizera, Ali Elbireer, Robert Lukande, Andrew Akampurira, Robert Wagubi, Henry Kajumbula, Grace Najjuka, Catherine Nanteza, Mariam Namawejje, Mark Ssennono, Agnes Kiragga, Edward Mpoza, Reuben Kiggundu, Lillian Tugume, Kenneth Ssebambulidde, Paul Kirumira, Carolyne Namuju, Tony Luggya, Julian Kaboggoza, Eva Laker, Alice Namudde, Conrad Muzoora, Kabanda Taseera, Liberica Ndyatunga, Brian Memela, Busingye Noeme, Emily Ninsiima, James Mwesigye, Rhina Mushagara, David R. Boulware, Melissa A. Rolfes, Kathy Huppler Hullsiek, Darlisha A. Williams, Radha Rajasingham, Joshua Rhein, Melanie W. Lo, Kirsten Nielsen, Tracy L. Bergemann, Paul R. Bohjanen, James Scriven, Edward N. Janoff, Nicholas Fossland, Monica Rani, Renee Carlson, Kate Birkenkamp, Elissa Butler, Tami McDonald, Anna Strain, Darin Wiesner, Maximilian von Hohenberg, Ann Vogt, Grant Botker, Nathan Bahr, Kosuke Yasukawa, Jason V. Baker, Sarah Lofgren, Anna Stadelman, Ananta S. Bangdiwala, Charlotte Schutz, Friedrich Thienemann, Graeme Meintjes, Yolisa Sigila, Monica Magwayi, Leya Hassanally, Tihana Bicanic, Lewis J. Haddow

**Affiliations:** 1 Infectious Diseases Institute, College of Health Sciences, Makerere University, P.O.BOX 22418 Kampala, Uganda; 2 Division of Infectious Diseases and International Medicine, Department of Medicine, University of Minnesota, Minneapolis, MN USA; 3 Department of Medicine, School of Medicine, College of Health Sciences, Makerere University, Kampala, Uganda

**Keywords:** Patient public involvement, Community engagement, Social support, Cryptococcal meningitis, HIV, Clinical trials, Sub-Saharan Africa

## Abstract

**Background:**

Clinical trials remain the cornerstone of improving outcomes for HIV-infected individuals with cryptococcal meningitis. Community engagement aims at involving participants and their advocates as partners in research rather than merely trial subjects. Community engagement can help to build trust in communities where these trials are conducted and ensure lasting mutually beneficial relationships between researchers and the community. Similarly, different studies have reported the positive effects of social support on patient’s outcomes. We aimed to describe our approach to community engagement in Uganda while highlighting the benefits of community engagement and social support in clinical trials managing patients co-infected with HIV and cryptococcal meningitis.

**Methods:**

We carried out community engagement using home visits, health talks, posters, music and drama. In addition, social support was given through study staff individually contributing to provide funds for participants’ food, wheel chairs, imaging studies, adult diapers, and other extra investigations or drugs that were not covered by the study budget or protocol. The benefits of this community engagement and social support were assessed during two multi-site, randomized cryptococcal meningitis clinical trials in Uganda.

**Results:**

We screened 1739 HIV-infected adults and enrolled 934 with cryptococcal meningitis into the COAT and ASTRO-CM trials during the period October 2010 to July 2017. Lumbar puncture refusal rates decreased from 31% in 2010 to less than 1% in 2017. In our opinion, community engagement and social support played an important role in improving: drug adherence, acceptance of lumbar punctures, data completeness, rate of screening/referrals, reduction of missed visits, and loss to follow-up.

**Conclusions:**

Community engagement and social support are important aspects of clinical research and should be incorporated into clinical trial design and conduct.

**Trial registration:**

ClinicalTrials.gov number, NCT01075152 and NCT01802385.

## Plain English summary

Involving patients and the public in healthcare and scientific research is increasingly becoming more important. It aims at involving patients’ community and their advocates as partners in scientific research rather than merely study subjects. Similarly, social support given to patients has previously been shown to have positive effects on patient’s treatment outcomes. In this study we aimed to highlight the benefits of community engagement and social support in two clinical studies managing patients co-infected with HIV and cryptococcal meningitis. We engaged the patients and their caregivers using home visits, health talks, posters, music and drama. In addition, we provided social support through study staff individually contributing to provide funds for participants’ food, wheel chairs, imaging studies, adult diapers, and other extra tests or drugs that were not covered by the study budget. The benefits of this community engagement and social support were assessed. Lumbar puncture refusal rates decreased from 31% in 2010 to less than 1% in 2017. In our opinion, community engagement and social support played an important role in improving: drug adherence, acceptance of lumbar punctures, data completeness, rate of screening/referrals, reduction of missed visits, and loss to follow-up. We therefore conclude by saying that community engagement and social support are very important aspects of clinical and scientific research and should be incorporated into the initial clinical study design and conduct.

## Background

Uganda is found in sub-Saharan Africa (SSA) where the prevalence of human immunodeficiency virus (HIV) and HIV-related opportunist infections is high [1, 2]. However, there is significant progress in the prevention and treatment of HIV in SSA mainly due to the massive roll out of antiretroviral treatment (ART) and recommendation to treat all HIV positive patients regardless of CD4 T-cell count [3]. Cryptococcal meningitis accounts for 15–25% of all HIV-related deaths [4–7]. An estimated 4000 HIV infected Ugandans develop *Cryptococcus* infection annually in the absence of screening and pre-emptive treatment for asymptomatic cryptococcal infection, and the estimated overall annual HIV-related cryptococcal mortality in Uganda is nearly 2500 deaths per year [7]. However, there remains a low index of clinical suspicion for all fungal infections in Uganda [8, 9]. Clinical trials provide a platform for improving outcomes for HIV-infected individuals with cryptococcal meningitis [10–14].

Community engagement in scientific research is defined broadly as the many ways in which the activities and benefits of scientific research can be shared with the community [15]. It can help build trust in communities where scientific research is conducted, improve community support for clinical research and ensure lasting mutually beneficial relationships between researchers and the community. Community engagement in clinical research should be a two-way process involving interaction, exchange and listening, with the aim of mutual benefit. Ideally, community engagement should begin as early in the biomedical research or clinical trial process as possible [16–18]. However, it is never too early or too late to involve the patients and participant community in a clinical trial.

Social support is a multifaceted experience that involves voluntary associations and is comprised of formal and informal relationships with others [19]. In sub-Saharan Africa, the majority of clinical trials are conducted in infectious diseases [20], which are the commonest causes of morbidity requiring hospitalization. A large proportion of inpatients enrolled in these trials present late with advanced disease. Thus, study participants are usually very ill and need social support to cope with the burden of disease, pill burden and multiple study procedures, which may be invasive in nature. This kind of social support can also be extended to the caregivers who are usually traumatised psychologically by the suffering of the patients. A few studies have reported the positive effects of social support on disease prognosis [21, 22] and similarly, low social support has been associated with worse treatment outcomes in different patient populations [19, 23].

In this article, we retrospectively describe the impact and benefits we have observed by actively engaging the patients’ and health workers’ communities together with providing extra social support during two prospective multi-site randomized clinical trials, which enrolled patients co-infected with HIV and cryptococcal meningitis in sub-Saharan Africa.

## Methods

Our Meningitis Clinical Research Team based at the Infectious Diseases Institute (IDI) Kampala Uganda, is dedicated to reducing advanced HIV-associated mortality by improving the diagnosis and management of common opportunistic infections, including meningitis. From October 2010 to July 2017, we conducted two large clinical trials during which we participated in community engagement and provided social support to study participants and their caregivers outside the usual study protocols. The Cryptococcal Optimal ART Timing (COAT) trial (ClinicalTrials.gov number, NCT01075152.), was conducted between 2010 and 2012. This randomised multi-centre clinical trial conducted in Uganda at Mulago National Referral Hospital, Mbarara regional referral hospital and Cape Town in South Africa evaluated the optimal timing of antiretroviral therapy (ART) initiation among patients with cryptococcal meningitis [11]. The results from this trial led to the current World Health Organization (WHO) recommendation that ART-naïve patients with cryptococcal meningitis should initiate ART 4–6 weeks following diagnosis and treatment of cryptococcal meningitis [24]. The second trial was the Adjunctive Sertraline for the Treatment of HIV-Associated Cryptococcal Meningitis (ASTRO-CM) study (ClinicalTrials.gov, number NCT01802385), a randomized multi-centre clinical trial conducted in Uganda at Mulago National Referral Hospital and Mbarara Regional Referral hospital between 2013 and 2017 [14]. This study evaluated the efficacy of adjunctive Sertraline as an antifungal drug for cryptococcal meningitis.

During both trials, we consented and screened 1739 HIV infected patients for meningitis and enrolled 934 patients. Herein, we discuss the benefits observed by actively engaging the patients, health workers and communities complemented by extra social support for trial participants. We followed the Guidance for Reporting Involvement of Patients and the Public (GRIPP2) checklist [25] (Additional file 1).

## Results and discussion

### Community engagement and its benefits

In these clinical trials, we were initially encouraged to engage the surrounding communities due to the high rate of refusal for lumbar punctures (25%) [26, 27], despite their diagnostic and therapeutic importance. Both clinical trials involved regular lumbar punctures performed to diagnose cryptococcal meningitis; performed at days 0,3,7,10, and 14 of treatment and as clinically indicated. In many African settings, there are many myths surrounding lumbar punctures. The majority of patients and/or their caregivers believed that lumbar punctures increased one’s chances of dying prematurely. We registered a 31% (*n* = 177) refusal rate in our first cohort [11] and this delayed diagnosis and prolonged the screening consenting process.

**Fig. 1:**
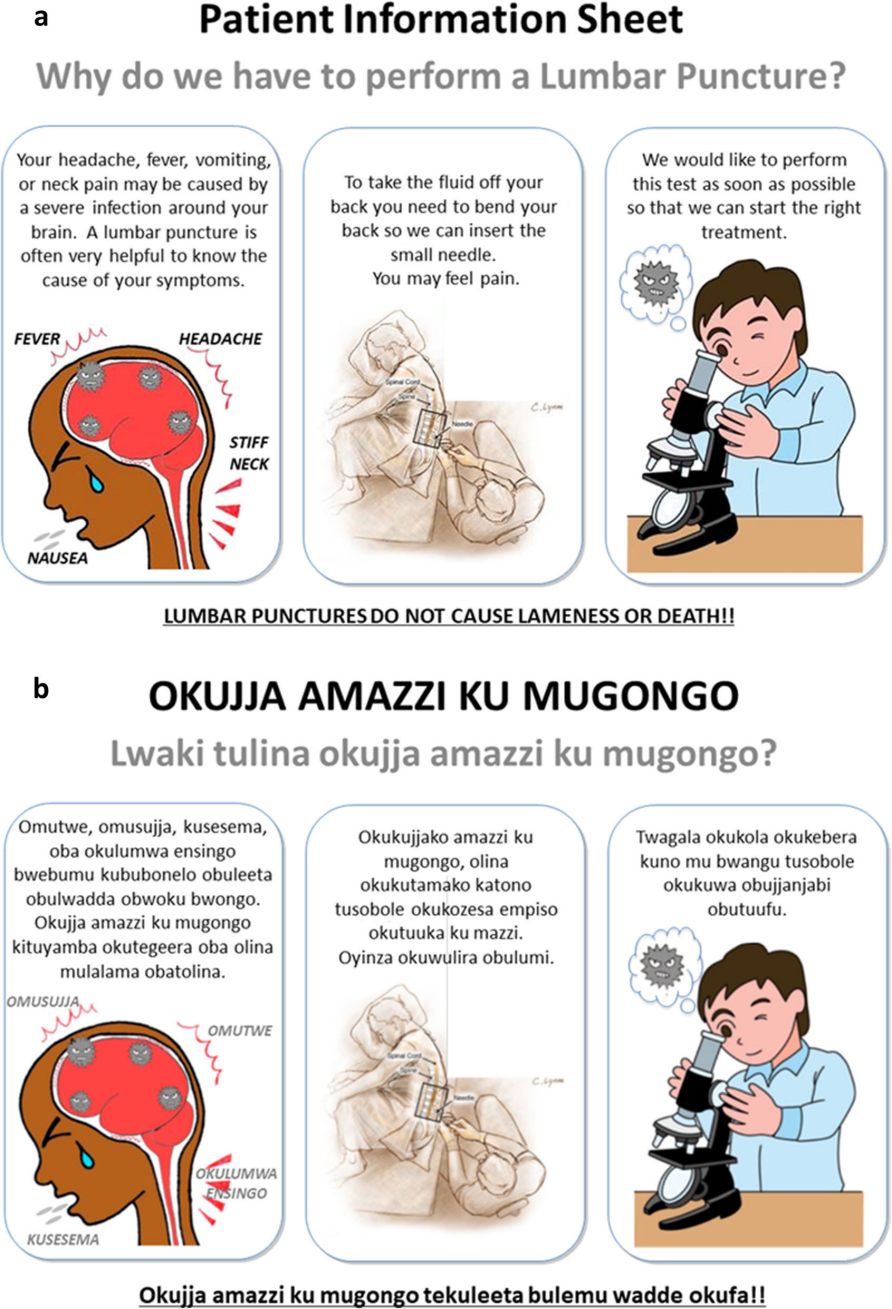
Poster for lumbar puncture procedure. **a)** English version, **b)** Luganda version

In an effort to reduce on the refusal rate for lumbar punctures, we initially made posters about lumbar punctures in English with additional translation into the local language (Luganda) (Fig. 1). The posters had a cartoon depiction of the lumbar puncture procedure, details on its importance, and indications for the procedure. These were placed on the infectious diseases and neurology wards. Other copies were given as handouts to the intern doctors rotating on these wards. This increased awareness about lumbar punctures and our research study among this community of health care providers and potential participants.

We then engaged the health workers (doctors, nurses and interns) about the clinical study we were conducting to raise awareness and get more referrals for study participants. In addition, we went out and sensitized more health care workers in other health centers and hospitals about cryptococcal meningitis and the clinical trial that was underway to encourage referrals. We conducted training focused on cryptococcal antigen (CrAg) screening in these peripheral centers. This engagement increased the referrals we received from peripheral health centers that could not perform lumbar punctures from zero (0) to 17 patients per month.

On a weekly basis, one of the nurses would gather all available caregivers for study participants on the ward and conduct health education. These talks emphasized the care of inpatients including patients’ hygiene, nutrition, ambulation, proper feeding, medication, and infection control during hospitalization and post discharge [10]. The caregivers were also reminded about the study procedures and their rights as regards the study. It is on the same platform that the myths about lumbar punctures were discussed and dispelled. We encouraged them to freely ask any questions about the study, myths and patient care. This helped to improve patients’ hygiene, nutrition and infection control during admission. Those caregivers that had understood what had been discussed were co-opted to share with other potential participants their own experience. These efforts also helped to increase the acceptance rate for lumbar punctures and recruitment rate.

In relation to the above, we also selected leaders among the patients’ caregivers to encourage others on drug adherence by reminding them to take their drugs on time. The leaders also helped patients who did not have caregivers to pick their drug prescriptions from the pharmacy and food from the kitchen staff. This effort helped to retain patients in care and improved adherence and treatment outcomes without missing any doses.

When participants missed several scheduled clinic visits (at least 3), and were uncontactable by phone, the team conducted a home visit for those who consented to home visits. During these home visits, we engaged the patient’s family members and available neighbors and discussed issues related to HIV stigma, myths, holistic care, study procedures and any other concerns the participant had about the study while emphasizing the need to keep all clinic appointments and adhere to prescribed medications. In addition, during these visits, we collected more contacts from other individuals whom the participant recommended, who could easily be contacted in case the participant could not be reached.

We also created a short movie about the importance of lumbar punctures in diagnosing meningitis (https://www.youtube.com/watch?v=222eM9s7xq0). This 20-min film was shown to patients, caregivers and health workers in order to increase awareness and decrease stigma around lumbar punctures. It so far has 96 views on YouTube. However, we estimate that more than 1000 people viewed the video in the places/clinics where it was screened. More recently, we conducted a community engagement event during which we educated the community about meningitis through music and drama. The music and drama events were aimed at engaging the hard to reach young adults who may be disconnected from HIV care in order to raise awareness about HIV, meningitis, the safety of lumbar punctures and ongoing meningitis clinical trials. The series of community engagement events we held were aimed at giving the active clinical research participants and those that successfully completed the trials a voice in sharing their experiences of clinical research and messages of hope around advanced HIV disease with the community, dispelling myths and stigma around HIV, raising awareness about the complications of advanced HIV disease and local ongoing clinical research and recent scientific advances. We also provided our phone contacts to the patients and so they would call us for free consultation even after the end of the trial.

### Social support and its benefits

For each study participant, we provided transport reimbursement for each scheduled outpatient clinic and sick visit. The amount was guided by ethics committee approval, thus this was not unique to our trial. However, in our study, most of our patients were very sick and required a number of caregivers to escort them for the clinic visit. This meant that we had to provide transport reimbursement for the patient and all accompanying caregivers. This would encourage the caregivers to promptly bring the patients for their clinic visits without fail. In addition, the majority of patients resided in rural areas far away from the clinic. This meant that the transport reimbursement tended to be higher than the pre-determined amount. Since we contacted the participants the day before each clinic visit, some informed us that they did not have any money to transport them for their return clinic visit. In such cases, we disbursed the money to them via “a mobile money” platform to ensure that appointments were kept.

Since the majority of participants were referred from rural areas, some had significant financial challenges and were unable to afford meals yet they had to take multiple medications including, but not limited to ART, antifungals, painkillers and multivitamins. In such cases, we provided financial support for food that came from a pool of donated money from study staff. This would help to avoid missing medications and improve adherence despite the high pill burden. Similarly, we partially supported participants to find accommodation to ensure that clinic visits were not missed when due. After the study period, we facilitated the referral of some participants to clinics that were nearest to where they were relocating. Occasionally, study staff collected to a small ‘social responsibility fund’ to contribute to food, wheel chairs, imaging studies, adult diapers, and other extra investigations or drugs that were not covered by the study budget while also helping some participants to start up small income generating businesses to take care of their own food, rent and other basic needs. In addition, study staff would donate clothes and shoes to the very needy patients. This encouraged the participants to remain in the study while building close relationships with study staff.

There was a proportion of study participants who had no caregivers at all. These are usually brought to hospital by either relatives, neighbors or “good Samaritans” and then abandoned. Since they are usually very sick and disoriented, we hired social workers to take care of such patients. In the Ugandan context, a medical social worker is one that is responsible for the emotional, social and economic welfare of patients admitted in the hospital and those brought to the hospital without close relatives including abandoned children. In cooperation and participation with health workers, the social worker investigates the family situations traumas and worries of patients who appear helpless and those unable to meet their health and medical care costs. They follow up complicated cases and give necessary advice to the health workers, Hospital Administration and the patients or their relatives. In our case, the social workers helped to pick their drug prescriptions from the pharmacy and food from the kitchen staff. They also cleaned up the patients and their beddings, and fed them as needed. They ensured that all medications were taken on time. However, in the absence of the social workers, all this would be done by the study nurses. This helped such patients to adhere to study medication, avoid nosocomial infections and eventually getting lost to follow-up.

A few of the study participants did not have mobile phones yet we needed to contact them for reminders about their clinic visits. These were mostly those without caregivers whose contacts would be taken as an alternative. In such cases, we bought basic mobile phones for the patient so as to stay in touch for follow up. These phones would also help us obtain verbal autopsies for participants who died at home. We would also send mobile money on these phones in case the patient needed transport to bring them for the clinic visit. Calls were made more often to these patients without caregivers to ensure adherence to all drugs.

Home visits were recommended when a patient missed two clinic visits and could not be reached by phone. As discussed above, families of the patients were engaged during home visits and discussed issues pertaining to care of participants and the relevance of attending clinic visits. The visiting counselor probed the participant to understand why visits were missed and found ways of troubleshooting to avoid them in future. In addition, during these visits, we collected more contacts from other individuals/next-of-kin who could easily be contacted in case the participant could not be reached.

Some funders of clinical trials have limitations to how much extra support you can give a participant beyond the routine clinical and lab investigations directly connected to the trial outcome. However, it should be noted that the social support provided was not used as an unethical means of persuading the participants to accept preceding medical or study procedures. Besides it was entirely voluntary on the side of the health workers and contributions were made after participants had been enrolled in the trials.

### Limitations of the study

The benefits presented in this paper are based on our experiences in Kampala during two longitudinal multisite HIV trials. The experiences may not be applicable in all resource-limited settings due to variations in socio-economic factors. Many of the observations made in this article are qualitative observations that lack quantitative outcome data. The reason for this paper is to highlight the importance of community engagement and social support to clinical trials, but trials specifically designed to evaluate the outcomes are lacking. These trials are needed to help inform future trial design about the most impactful and cost-effective interventions.

## Conclusion

Community engagement has increasingly become an important aspect of scientific research and is currently encouraged by many funders as part of research grant applications. It creates opportunities for dialogue and encourages the research participants to fully engage in the study when they understand it well. Social support as described in this paper is an aspect that has not been applied in many clinical studies and therefore the extent of its benefits is unclear. However, with these two strategies, we observed many benefits that we believe contributed to the minimal loss to follow up during these trials. They helped us to improve adherence, acceptance of lumbar punctures (from 31% to less than 1%), data completeness, rate of screening and referrals. In addition, we managed to minimize missed visits and attrition of participants.

## Supplementary information

**Additional file 1.** GRIPP2 checklist. File contains a GRIPP2 checklist indicating pages, which report the information that meets the criteria of the checklist.

## Data Availability

All data generated or analysed during this study are included in this published article and its supplementary information files. The authors confirm that all data underlying the findings are fully available without restriction and can be availed by contacting Mr. Richard Kwizera (kwizerarichard@ymail.com).

## Abbreviations

ART: Antiretroviral therapy
ASTRO-CM: Adjunctive Sertraline for the Treatment of HIV-Associated Cryptococcal Meningitis
COAT: Cryptococcal Optimal ART Timing
CrAg: Cryptococcal antigen
HIV: Human immunodeficiency virus
UNCST: Uganda National Council of Science and Technology
WHO: World Health Organization
