# Supplementary material for: Impact of community engagement and social support on the outcomes of HIV-related meningitis clinical trials in a resource-limited setting
Source: Res Involv Engagem. 2020 Aug 20;6:49. doi: 10.1186/s40900-020-00228-z (PMC7441728; doi:10.1186/s40900-020-00228-z)
Supplement: Supplementary file 1 — Additional file 1. GRIPP2 checklist. File contains a GRIPP2 checklist indicating pages, which report the information that meets the criteria of the checklist. [file 40900_2020_228_MOESM1_ESM.docx]

| **Section and topic** | **Item** | **Reported on page No** |
| --- | --- | --- |
| 1: Aim | Report the aim of PPI in the study | Page 2  line 25-27 |
| 2: Methods | Provide a clear description of the methods used for PPI in the study | Page 5  Line 98-116 |
| 3: Study results | Outcomes—Report the results of PPI in the study, including both positive and negative outcomes | Page 6  Lines 117-219 |
| 4: Discussion and conclusions | Outcomes—Comment on the extent to which PPI influenced the study overall. Describe positive and negative effects | Page 6  Lines 117-237 |
| 5: Reflections/critical perspective | Comment critically on the study, reflecting on the things that went well and those that did not, so others can learn from this experience | Page 11  Lines 220-237 |
